# COMPARING INFORMANT BASED MEASURES OF COGNITIVE FUNCTIONING BETWEEN THE US AND MEXICO

**DOI:** 10.1093/geroni/igac059.2994

**Published:** 2022-12-20

**Authors:** Yuan Zhang, Phillip Cantu, Joseph Saenz

**Affiliations:** University of North Carolina at Chapel Hill, Chapel Hill, North Carolina, United States; The University of Texas Medical Branch, Galveston, Texas, United States; University of Southern California, Los Angeles, California, United States

## Abstract

Informant reports are important for understanding cognitive impairment and dementia in older populations. Recently, several population-based aging studies have included harmonized direct and informant assessments to assess the prevalence of dementia globally as part of the international Harmonized Cognitive Assessment Protocol (HCAP). However, the social and cultural contexts of nations, sociodemographic characteristics of informants, and the subject-informant relationship may affect the meaning and accuracy of informant reports of cognitive decline and functioning. This study examines the extent to which informant characteristics and the informant-respondent relationships are associated with informant-reported cognitive function in the United States and Mexico. Data are from the 2016 US Health and Retirement Study (HRS) HCAP (Nf 2,918) and the Ancillary Study of Cognitive Aging in Mexico (Mex-Cog) (Nf 1,750). Informant-reported cognitive function was measured by the informant Community Screening Instrument for Dementia (CSI-D). Linear regression is used to assess the association of informant characteristics and the information-participant relationship with the total CSI-D score. We find older Americans have worse informant-reported cognitive functioning but higher directly-assessed cognitive functioning than Mexicans. Nearly 80% of informants lived with the subject in Mexico, compared to less than half in the US. In both countries, older informants, children and other family members (compared to spouse) report less cognitive impairment. In Mexico, female informants reported more impairment. In the US, coresident informants reported more impairment. This research shows how social environments influence informant reporting. Understanding heterogeneity in informants is vital when examining informant measures of cognitive function in cross-countries studies.

